# Glioblastoma-derived exosomes promote lipid accumulation and induce ferroptosis in dendritic cells via the NRF2/GPX4 pathway

**DOI:** 10.3389/fimmu.2024.1439191

**Published:** 2024-08-13

**Authors:** Jian Yang, Mingqi Zhang, Xuying Zhang, Yue Zhou, Tingting Ma, Jia Liang, Jinyi Zhang

**Affiliations:** ^1^ Institution of Life Science, Jinzhou Medical University, Jinzhou, China; ^2^ Liaoning Provincial Key Laboratory of Neurodegenerative Diseases, Jinzhou Medical University, Jinzhou, China; ^3^ Liaoning Technology and Engineering Center for Tumor Immunology and Molecular Theranotics, Jinzhou Medical University, Jinzhou, China; ^4^ Oujiang Laboratory (Zhejiang Lab for Regenerative Medicine, Vision and Brain Health), Wenzhou, China

**Keywords:** glioblastoma, dendritic cells, exosomes, ferroptosis, lipid accumulation

## Abstract

Glioblastoma-derived exosomes (GDEs), containing nucleic acids, proteins, fatty acids and other substances, perform multiple important functions in glioblastoma microenvironment. Tumor-derived exosomes serve as carriers of fatty acids and induce a shift in metabolism towards oxidative phosphorylation, thus driving immune dysfunction of dendritic cells (DCs). Lipid peroxidation is an important characteristic of ferroptosis. Nevertheless, it remains unclear whether GDEs can induce lipid accumulation and lipid oxidation to trigger ferroptosis in DCs. In our study, we investigate the impact of GDEs on lipid accumulation and oxidation in DCs by inhibiting GDEs secretion through knocking down the expression of Rab27a using a rat orthotopic glioblastoma model. The results show that inhibiting the secretion of GDEs can reduce lipid accumulation in infiltrating DCs in the brain and decrease mature dendritic cells (mDCs) lipid peroxidation levels, thereby suppressing glioblastoma growth. Mechanistically, we employed *in vitro* treatments of bone marrow-derived dendritic cells (BMDCs) with GDEs. The results indicate that GDEs decrease the viability of mDCs compared to immature dendritic cells (imDCs) and trigger ferroptosis in mDCs via the NRF2/GPX4 pathway. Overall, these findings provide new insights into the development of immune-suppressive glioblastoma microenvironment through the interaction of GDEs with DCs.

## Introduction

Glioblastoma is the most prevalent malignant primary brain tumor, making up about 51.4% of such tumors. Its median survival is 14.6 months, with a 5-year survival rate of just 4–5% (1). In the tumor microenvironment, exosomes derived from tumors play an important role. It is a cellular vesicle the size of 30–150nm.Tumor cells release exosomes that can communicate with nearby and distant cells, influencing them through cell surface receptor interactions or by being taken up through endocytosis (2, 3). In glioblastoma, glioblastoma-derived exosomes (GDEs) are crucial for promoting glioblastoma cell proliferation, angiogenesis, metastasis, and accelerating epithelial to mesenchymal transition (4–6). Additionally, GDEs have been found to suppress immune system function (7, 8).

Dendritic cells (DCs) are crucial antigen-presenting cells (APCs) that initiate primary T-cell responses (9, 10). Tumor-derived exosomes have been shown to suppress the immune system, particularly by inhibiting DC differentiation, maturation, and function (11). These exosomes can also transport fatty acids, impacting DC lipid metabolism and potentially elevating lipid levels in tumor-infiltrating DCs (12). High lipid loads in DCs can impair antigen processing and presentation, as well as hinder MHC class I complex transport to the cell surface, thereby negatively modulating the antitumor immune response and promoting tumor growth (13).

The accumulation of fatty acids can lead to lipotoxicity by causing mitochondrial damage, endoplasmic reticulum stress, and disrupting membrane integrity (14–17). It can also promote lipid peroxidation, leading to ferroptosis. Ferroptosis is a novel type of programmed cell death that is iron-dependent and distinct from apoptosis, necrosis, and autophagy (18). Morphological characteristics include reduced volume of mitochondria, increased bilayer density, reduction or absence of mitochondrial ridges, and smaller cells separated from one another (19–21), while biochemical characteristics include glutathione depletion, reduced activity of glutathione peroxidase 4 (GPX4), and Fe^2+^ which generate large amounts of reactive oxygen species in a Fenton reaction, contributing to lipid peroxidation and causing ferroptosis (22, 23). The nuclear factor E2-related factor (NRF2) was discovered to be an important antioxidant protein. In glioblastoma research, it has been found inhibiting the expression of NRF2 in DCs promote their maturation, leading to a decrease in their antioxidant capacity (24). NRF2 can modulate ferroptosis by regulating the synthesis of GPX4 through the glutamate-cystine transport system (systemxc-) in the pathway (25). In this study, we investigated whether GDEs enhance lipid accumulation in glioblastoma-infiltrating DCs and triggers ferroptosis in DCs using both *in vivo* and *in vitro* glioblastoma models.

## Materials and methods

### Cell lines

Rat CTX astrocyte cells (Geneio Bio, Guangzhou) and C6 glioblastoma cells (Shanghai Academy of Life Sciences, Chinese Academy of Sciences) were cultured in DMEM (Gibco, cat: 8121669) supplementsed 10% FBS (AusgeneX, cat: C0227), 100 U/mL penicillin and 100 U/mL streptomycin at 37°C, 5% CO_2_.

### Animals and glioblastoma model

Male Sprague Dawley rats (6–8 weeks old) were obtained from Jinzhou Medical University’s Experimental Animal Center. C6 cells were injected into the right lateral ventricle of each rat at concentration 5×10^5^ cells/10 μL. A hole was drilled in the exposed skull (stereotactic coordinates: 1 mm anterior to bregma and 3 mm to the right of sagittal suture). The injection needle was inserted into the hole to a depth of 6 mm. The needle was then withdrawn by 1 mm. The injection was performed at a rate of 1 μL/min, after completing the injection, the needle was left in place in the brain for an additional 2 min to minimize the potential leakage of injected fluid. Finally, the hole was sealed with dental glue and sutured accordingly.

### BMDCs culture

Male SD rats (160–220 g) were anesthetized with isoflurane. The tibia and femur were aseptically cut off at the ends and separated. The bone ends were removed using ophthalmic scissors, and the bone marrow cavity was flushed with 7 mL of PBS using a syringe. To ensure purity, the resulting suspension was then filtered through a 200 μm filter and centrifuged in an equal volume of Ficoll isolate. The cells were adjusted to a concentration of 1×10^6^/mL and cultured in 6-well plates with RPMI 1640 medium (Gibco, cat: 8120343) containing 10% FBS, 20 ng/mL recombinant rat granulocyte-monocyte colony stimulating factor (RRGM-CSF) (Absin, cat: abs01067), and 20 ng/mL recombinant rat interleukin 4 (RRIL-4) (Absin, cat: abs01053) in a cell incubator set to 37°C with 5% CO_2_. ImDCs were harvested at 6 days, and mDCs were obtained by adding 50 ng/mL of recombinant rat tumor necrosis factor-alpha (RRTNF-α) (Absin, cat: abs04633) to the immature DCs and culturing them for 48 h (26, 27).

### Western blot analysis

Cells were lysed on ice in RIPA buffer (Solarbio, cat: R0010) supplemented with PMSF lysis buffer (Cat.No.P0100, Solarbio, China). The mixture was centrifuged at 12,000 rpm at 4°C for 15 min to collect the supernatant containing proteins. Proteins samples were separated by SDS-PAGE. Subsequently, the proteins were transferred onto PVDF membranes, which were probed with specific primary antibodies. The antibodies used were: ALIX antibody (Proteintech, cat:12422–1-AP; 1:1000), TSG101 antibody (abcam, cat:ab125011; 1:2000), Calnexin antibody (Bioworld, cat:cj36131; 1:700), NRF2 antibody (Cell Signaling, cat:336493, 1:2000), GPX4 antibody (Abcam, cat:ab125066, 1:2000), SLC7A11 antibody (Abcam, cat:ab175186, 1:2000), Rab27a antibody (Zen-Bioscience, cat:340884, 1:1000), β-actin antibody (Cell Signaling, cat:3700, 1:2000). The membrane was incubated with the primary antibodies at 4°C overnight, followed by probing with horseradish peroxidase-conjugated secondary antibodies. Finally, protein bands were visualized using enhanced chemiluminescence reagents.

### Exosome purification and characterization

C6 cells and CTX cells were replaced at 70% growth with EXO-free 10% FBS (Gibco/centrifuged to remove exosomes) in the DMEM medium. C6 cells and CTX cells were replaced at 70% growth with EXO-free 10% FBS (Gibco/centrifuged to remove exosomes) in the DMEM medium. After 48 h, the cell cultures were collected and centrifuged at 400 g for 10 min, 2000 g for 10 min, and 10000 g for 30 min to remove cell debris, filtered through a 0.22 μm filter, and then used. The exosomes were collected by centrifugation at 100,000 g for 80 min (Beckman, Optima XPN-100 Ultracentrifuge), and the exosome concentration was determined using a BCA protein assay kit (Thermo, cat: 23228). Western blot analysis was used to determine the expression of the exosome-specific markers ALIX, TSG101, and Calnexin. The concentration of exosomes was determined by NanoSight Tracking Analysis (NTA). Transmission electron microscopy (Hitachi, HT-7700) was used to confirm the exosome morphology according to references (28, 29).

### Flow cytometry analysis

To prepare a single-cell suspension for staining, brain tissue was minced and then digested it with 0.25% trypsin for 20 min. After digestion, the mixture was filtered through a 70μm strainer and centrifuged at 2000 rpm for 30 min at room temperature using Procell (Solarbio, cat: P8370). Similarly, the spleen was minced to obtain a single-cell suspension, the cell suspension was added to Ficoll-Paque Plus at 400g for 30 min at room temperature (30). Single-cell suspensions of 1×10^6^ cells were stained with flow antibodies, specifically anti-CD4 (BioLegend, cat:201509; 1:100), CD3 (BioLegend, cat:201403; 1:100), CD8 (BioLegend, cat:200607; 1:100), MHCII (BioLegend, cat:205305; 1:100), CD103 (BioLegend, cat:205505; 1:100), CD80 (BioLegend, cat:200205; 1:100), CD86 (BioLegend, cat:200314; 1:100), CD45 (BioLegend, cat:202226; 1:100) cell surface marker. Flow cytometry was performed using an LSR II flow cytometer (BD Biosciences) and analyzed using FlowJo software version 10.

### Immunohistochemical staining

The brain sections were deparaffinized, hydrated, and incubated with 3% H_2_O_2_ for 10 min to block endogenous peroxidase activity. Subsequently, the sections were subjected to high pressure for 10 min while being immersed in a prewarmed antigen retrieval solution (0.01 M, pH 6.0, citrate buffer). Following that, the brain sections were sealed with 5% bovine serum albumin and incubated overnight at 4°C with primary antibodies Ki67 (Abcam, cat: ab16667; 1:200) and CD103 (Abcam, cat: ab224202; 1:200). Biotin-coupled anti-rabbit antibodies and streptavidin-biotin were then applied. DAB horseradish peroxidase color development kit (ZSGB-bio, cat: ZLI-9018) was utilized to visualize the brain tissue sections, which were subsequently counterstained with hematoxylin, and observed under a microscope.

### HE staining

The brain tissues were immobilized in 10% neutral buffered formalin for a duration of 24 h. Subsequently, sections measuring 5 μm in thickness and composed of paraffin-embedded tissues were subjected to hematoxylin and eosin staining (Solarbio, cat: G1120). The analysis of the brain sections for pathology grading was conducted by an experienced pathologist.

### CCK-8, LDH, and ELISA tests

GDEs (160 μg/mL) were administered to DCs for 24 h. The ability of DCs to secrete IL-12 was evaluated in accordance with the ELISA kit’s (Solarbio, cat: SEKR0057) manufacturer’s instructions. The manufacturer’s instructions for the cell viability kit were followed when conducting the LDH (Progema, cat: G1781) and cell viability assays (SEVEN, cat: SC119–01).

### Rab27a knockdown

Short hairpin RNAs (shRNA) targeting Rab27a and Control scrambled shRNA were designed (Genomeditech, Shanghai, China), Vector information is as follows (PGMLV-HU6-MCS-CMV-ZsGreen1-PGK-Puro). (NC target sequence TTCTCCGAACGTGTCACGT, sh1 target sequence GGAAGTTCAACTCCAAATTCA, sh2 target sequence GCCTGACCACGGCATTCTTCA, sh3 target sequence GGATAAGCCAGCTACAGATGC). We transfected 293T cells with 5 μg of plasmid (PGMLV-SC5:psPAX2:pMD2.G=4:3:1) and 7.5 μL of Lipofectamine 3000. The medium containing lentivirus was harvested at 48 h and added to the C6 cells. After 72 h, the cells were observed under an inverted phase contrast fluorescence microscope for green fluorescent protein expression, puromycin (1 μg/mL) was added to screen out poorly transfected cells, and Western blot was used to detect the transfection efficiency of each group.

### ROS assay

DCs were given GDEs, and they were then kept in an incubator overnight at 37°C with 5% CO_2_. Remove the supernatant. Then, add the highly sensitive DCFH-DA dye working solution (Dojindo, cat: R252) to the DCs and incubate them at 37°C for 30 min in an incubator with 5% CO_2_. The working solution was removed, washed twice with HBSS, centrifuged for wall attachment (Thermo, Cytospin4), the fluorescence intensity was observed under fluorescence microscope.

### GSH and MDA assays

After a 24 h treatment with GDEs (160 μg/mL), glutathione and malondialdehyde in the DCs were assessed using the GSG/GSSG assay kit (Beyotime, cat: S0053) and MDA assay Kit (Dojindo, cat: M496). In each group, there were at least three replications.

### Liperfluorescence assay

DCs were treated with GDEs (160 μg/mL) for 24 h. The culture medium was removed, and the cells were washed twice with 200 μL of HBSS. Following medium removal, a 200 μL solution of Liperfluo (Dojindo, cat: L248) diluted with 1 μmol/L HBSS was added. The solution was incubated with controlled temperature (37°C) and 5% CO2 for 30 min. Two washes were performed using 200 μL of HBSS following the removal of the solution. Fluorescence intensity was evaluated using fluorescence microscopy.

### Fe^2+^ assay

To initiate the experiment, inoculate 96-well clear-bottomed black plates with 100 μL of DCs at a concentration of 20,000 cells per well. Treat the cells with EXO and incubate them overnight at 37°C in a 5% CO_2_ incubator. Subsequently, wash all the wells three times with 100 μL of Hank’s Balanced Salt Solution (HBSS). Next, add 100 μL of a working solution of FerroOrange (Dojindo, cat: F374) with a concentration of 1 μmol/L and incubate for 30 min at 37°C in a 5% CO_2_ incubator. Finally, utilize a multifunctional microplate reader to measure the fluorescence intensity of each sample.

### JC-1 assay

DCs were treated with GDEs (160 μg/mL) for 24 h. Mitochondrial membrane potential changes were detected using a kit (Solarbio, cat: M8650).

### Statistical analysis

All data were presented as the mean ± SD (standard deviation) of at least three independent experiments. Student’s t-test and one-way analysis of variance (ANOVA) were used to assess the significance of the differences using GraphPad Prism 5. Data were visualized using GraphPad Prism version 5.0 software, and *P<* 0.05 was considered statistically significant.

## Results

### Lipid accumulation is significantly increased in infiltrating DCs in the glioblastoma

**Figure 1:**
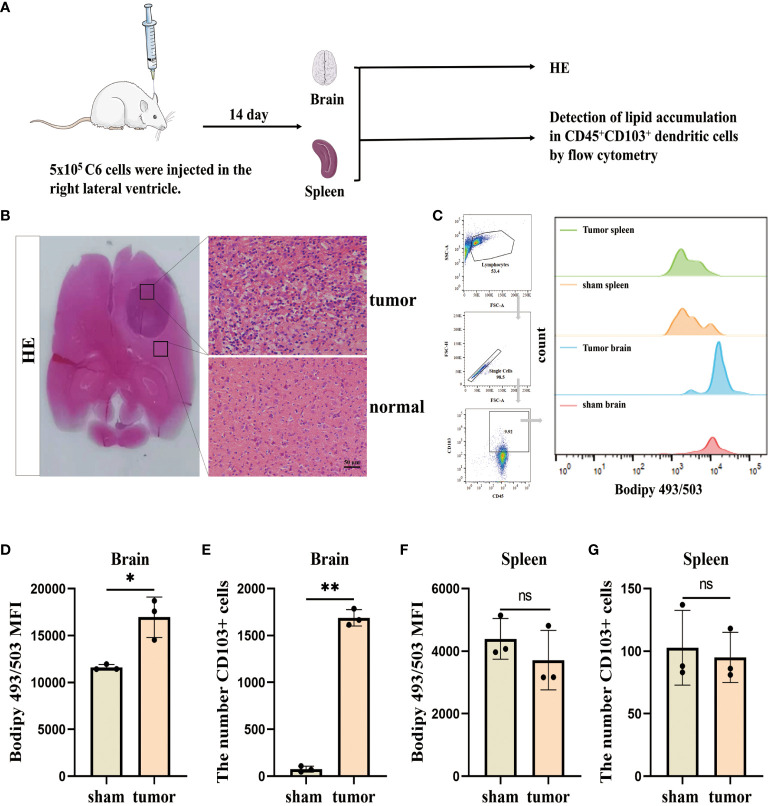
Lipid accumulation is significantly increased in infiltrating DCs in the glioblastoma. **(A)** This section describes the flow of the results manipulation process. **(B)** SD rats were injected with 5×10^5^ cells/10 µL of C6 cells into their right lateral ventricle. After a 14 d, the animals were euthanized and subjected to HE staining. Scale bars, 50 μm. **(C)** CD45^+^CD103^+^ DCs in the brain and spleen using BODIPY 493/503 lipid dye, and employ flow cytometry analysis and bar charts for visualization purposes. **(D–G)** Lipid content and CD45^+^CD103^+^ DCs number were counted in the brain **(D, E)** and spleen **(F, G)** with n = 3. Bars represent mean ± SD, *p<0.05; **p<0.01.

We initially assessed the lipid accumulation in DCs *in vivo*, and the experimental design involving animals is presented in (**Figure 1A**). To establish a glioblastoma model, C6 cells were injected into the right ventricle. HE staining revealed multiple lamellar irregular tumor lesions in the brain parenchyma of the rats. Tumor cells exhibited clear heterogeneity and show vigorous proliferation, consistent with the pathological manifestation of glioblastoma (**Figure 1B**). Subsequently, we isolated monocytes from the brain and spleens, and stained CD45^+^CD103^+^ DCs using Bodipy 493/503 dye (**Figure 1C**). Flow cytometry analysis demonstrated a high lipid content in the tumor group compared to the sham group (no C6 glioblastoma cells injection after drilling the right lateral ventricle) and an increased number of infiltrating CD45^+^CD103^+^ DCs in the brain (**Figures 1D, E**). Conversely, no significant changes were observed in the spleen (**Figures 1F, G**). These findings indicate the presence of lipid accumulation in infiltrating DCs in the glioblastoma.

### Inhibition of GDEs secretion reduces lipid accumulation glioblastoma-infiltrating DCs

**Figure 2:**
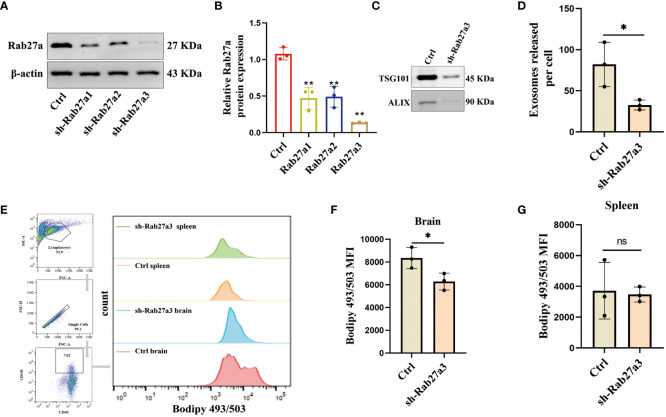
Inhibition of GDEs secretion reduces lipid accumulation and mitigates lipid peroxidation in glioblastoma-infiltrating DCs. **(A, B)** Western blot assay of the three knockdown targets with Rab27a to screen the most efficient target with n = 3. **(C)** Western blott is used to detect the changes in exosome-associated marker proteins ALIX and TSG101 after sh-Rab27a3 in glioblastoma cells, in order to assess the exosome content. **(D)** The number of exosomes released per cell (total number of exosomes/total number of cells) after sh-Rab27a3, NanoSight tracking analysis (NTA) was employed. **(E)** The CD45^+^CD103^+^ DCs in the brain and spleen of the Control and sh-Rab27a3 glioblastoma model were stained using the lipid dye BODIPY 493/503. Visualization is achieved through flow cytometry analysis and bar charts. **(F, G)** Statistical analysis of lipid fluorescence intensity of DCs in the brain **(F)** and spleen **(G)** of Control and sh-Rab27a3 glioblastoma model groups with n = 3. Bars represent mean ± SD. *p<0.05; **p<0.01.

To investigate the impact of GDEs on the lipid accumulation in glioblastoma-infiltrating DCs, we utilized sh-Rab27a to inhibit the secretion of GDEs in glioblastoma cells. Rab27a is a type of Ras small GTPase in the Rab family that plays a role in regulating the release of exosomes (31). Through Western blot analysis, we confirmed that sh-Rab27a3 displayed the highest interference efficiency (**Figures 2A, B**). We also examined the exosomal marker proteins (TSG101, ALIX) (**Figure 2C**) using NanoSight tracking analysis (NTA) to quantify the amount of exosomes released by C6 cells after sh-Rab27a3, scrambled shRNA was used as a negative Control. The results showed that sh-Rab27a3 significantly inhibited the secretion of exosomes in C6 cells compared to Control (**Figure 2D**). However, inhibiting GDEs secretion does not have obvious impact on the proliferation, invasion, and migration of glioblastoma cells *in vitro* (**Supplementary Figure 1**). Subsequently, we used C6 cells transfected with sh-Rab27a3 to establish a rat glioblastoma model, with a scrambled nonsense shRNA sequence was used as the Control. After 14 days tumor cell inplantation, the lipid content of CD45^+^CD103^+^ DCs in the brain and spleen of both groups was measured using Bodipy 493/503 immunofluorescence staining and flow cytometric analysis (**Figure 2E**). The results showed that the lipid content of DCs in the brain was lower in the sh-Rab27a3 group compared to the Control, while no significant differences were observed in DCs in the spleen (**Figures 2F, G**). These findings suggest that inhibiting GDEs secretion in glioblastoma tumor cells reduced lipid accumulation in glioblastoma-infiltrating DCs *in vivo*.

### GDEs promote lipid accumulation and cell death of DCs by directly transferring fatty acids

**Figure 3:**
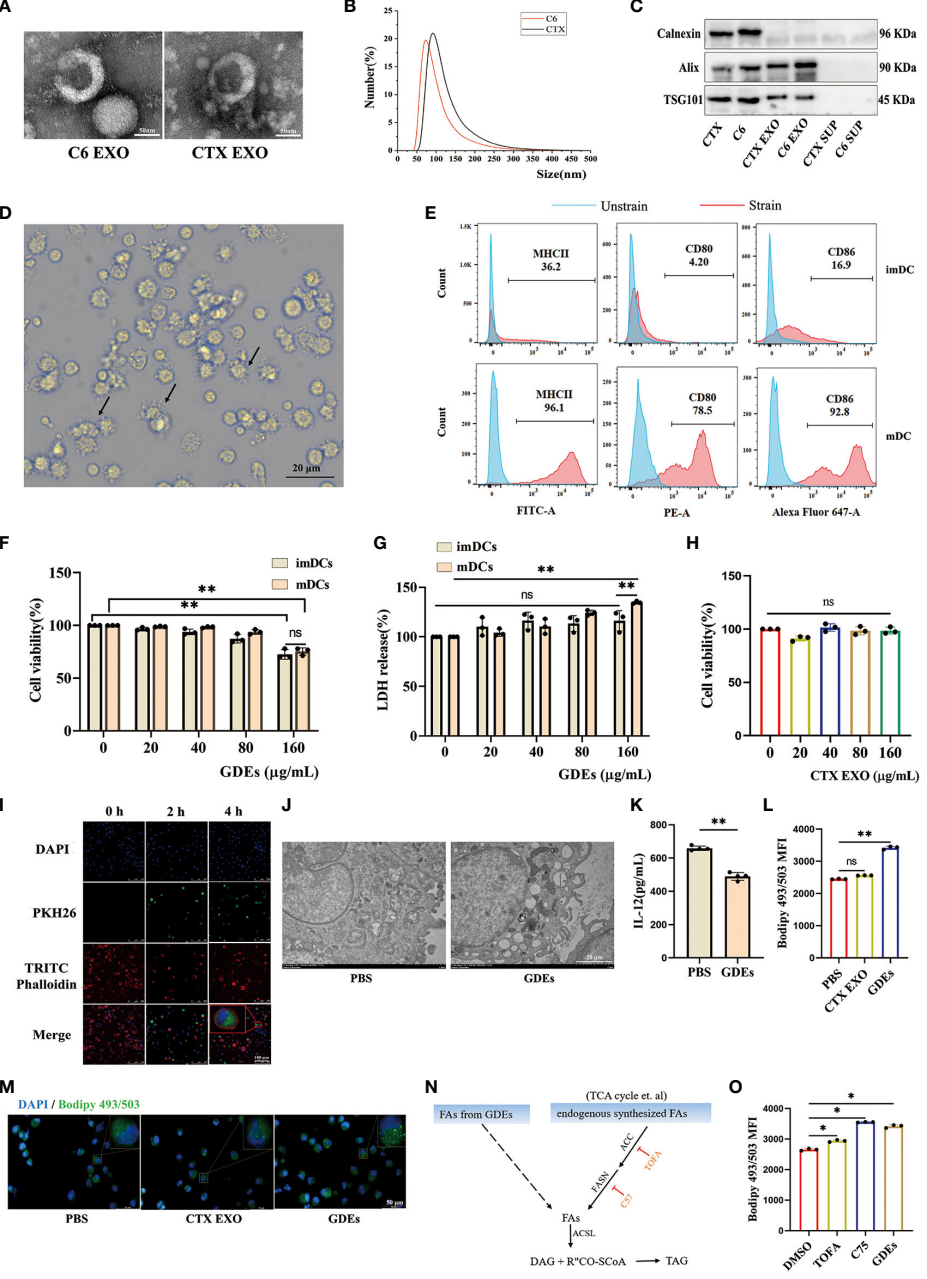
GDEs promote lipid accumulation and cell death of DCs by directly transferring fatty acids. **(A)** Exosome morphology in CTX and C6 cells as seen through a transmission electron microscope. Scale bars, 50 μm. **(B)** Exosomes from CTX and C6 cells had a diameter of approximately 100 nm, according to a particle size analysis. **(C)** Western blots analysis of exosomes-related proteins (Alix, Calnexin, TSG101) in CTX and C6 cells exosomes, CTX and C6 cells supernatant. **(D)** Photographs of DCs taken after 6 d of culture show the cells to have a protruding morphology. Scale bars, 20 μm. **(E)** The upper panel imDCs collected on 6 d. The lower panel shows mDCs obtained on 6 d after 48 h of exposure to 50 ng/mL TNFα. MHCII/CD80/CD86 expression was analyzed using flow cytometry. **(F, G)** Both imDCs and mDCs that were administered 0, 20, 40, 80, 160 μg/mL GDEs were cell viability **(F)** and LDH release level **(G)** assayed by CCK8 and LDH for 24 h with n = 3. **(H)** The administration of CTX EXO to mDCs at concentrations of 0, 20, 40, 80, 160 μg/mL had no impact on the results of the 24 h cell activity assay with n = 3. **(I)** GDEs labeling with PKH67, DCs nuclei stained with 355642 dyes, the DCs cytoskeleton labeled with TRITC phalloidin, and DCs uptake of GDEs observed by laser confocal microscopy (40× magnification), Scale bars, 100 μm. **(J)** Transmission electron microscopy (TEM) is used to observe the morphology of lipid droplets within mDCs. **(K)** The reduction in IL-12 secretion capacity was assessed by ELISA after mDCs were treated for 24 h with 160 μg/mL GDEs with n = 4. **(L, M)** mDCs were treated with CTX EXO and GDEs, and lipid content was observed by fluorescence microscopy **(M)** and quantified by flow cytometry **(L)**. **(N)** Schematic diagram of triglyceride synthesis. ACC, Acetyl-CoA carboxylase. ACSL, Long-chain acyl-CoA synthetase. DAG, Diacylglycerol. FASN, Fatty acid synthase. TAG, Triacylglycerol. **(O)** Under the influence of DMSO, TOFA (5 μM), and C75 (30 μM), the change in intracellular lipid levels of mDCs cultured with GDEs for 24 hours can be observed. Bars represent mean ± SD. *p<0.05; **p<0.01.

To investigate the mechanisms by which GDEs regulates lipid level and survival of glioblastoma-infilltrating DCs, we purified exosomes derived from glioblastoma cells and investigated their potential role in DCs. Initially, GDEs were isolated from the supernatant of C6 glioblastoma cells and CTX cells (astrocyte cell line) using ultracentrifugation. TEM analysis of exosomes revealed the presence of cup-like structures (**Figure 3A**). Particle size analysis indicated an approximate diameter of 100 nm for the particles (**Figure 3B**). Exosome-specific markers (Alix, calnexin, TSG101) were detected using Western blot analysis (**Figure 3C**). Bone marrow derived DCs (BMDCs) were obtained by *in vitro* culture of marrow cells. Flow cytometry analysis confirmed increased expression of MHC II, CD80, and CD86 in CD45^+^CD103^+^ mDCs, while the opposite pattern was observed in imDCs (**Figures 3D, E**). ImDCs and mDCs were exposed to GDEs at various concentrations (0, 20, 40, 80, and 160 µg/mL) for 24 h, their viability and LDH levels were assessed using CCK8 and LDH assay. CCK8 and LDH assays revealed concentration-dependent cell death in mDCs after 24-hour treatment with GDEs, with a more pronounced effect observed at 160 µg/mL (**Figures 3F, G**). Consequently, mDCs and GDEs at 160 µg/mL were selected for subsequent investigation. In contrast, various concentrations of CTX-derived EXO were administered to mDCs for 24 h. The CCK8 assay results demonstrated no significant effect on DCs survival (**Figure 3H**). Simultaneously. PKH67-labeled GDEs were co-cultured with mDCs. The green fluorescent signals within the cytoplasm of mDCs indicated the internalization of GDEs, which increased over time from 0h to 4 h (**Figure 3I**). We also observed that GDEs significantly caused lipid accumulation (**Figures 3J, L, M**) and decreased IL-12 secretion (**Figure 3K**) compared with CTX EXO. These data indicate that the reduction in mDCs cell viability and lipid accumulation was specific to GDEs, suggesting that GDEs treatment lead to increased lipid accumulation, reduced survival and compromised functions of mDCs.

In the tumor microenvironment, lipid accumulation in DCs is primarily characterized by triglycerides (32), which require fatty acids from two sources for synthesis: exogenous fatty acids (derived from GDEs) and endogenously synthesized fatty acids (**Figure 3N**). To investigate whether the lipid accumulation in mDCs treated with GDEs was the result of taking up fatty acids from GDEs or a result of GDE-induced denovo synthesis, specific inhibitors were used to block key steps in the *de novo* synthesis pathway, such as ACC (acetyl-coenzyme A [CoA] carboxylase) inhibitor 5-tetradecyloxy-2-furanone (TOFA) and fatty acid synthase (FASN) inhibitor C75. TOFA and C75 did not reduce the increase in lipid content induced by GDEs (**Figure 3O**). Therefore, it can be concluded that lipid accumulation in mDCs is the result of directly taking up fatty acids from GDEs.

### GDEs induced DCs ferroptosis via NRF2/GPX4 pathway

**Figure 4:**
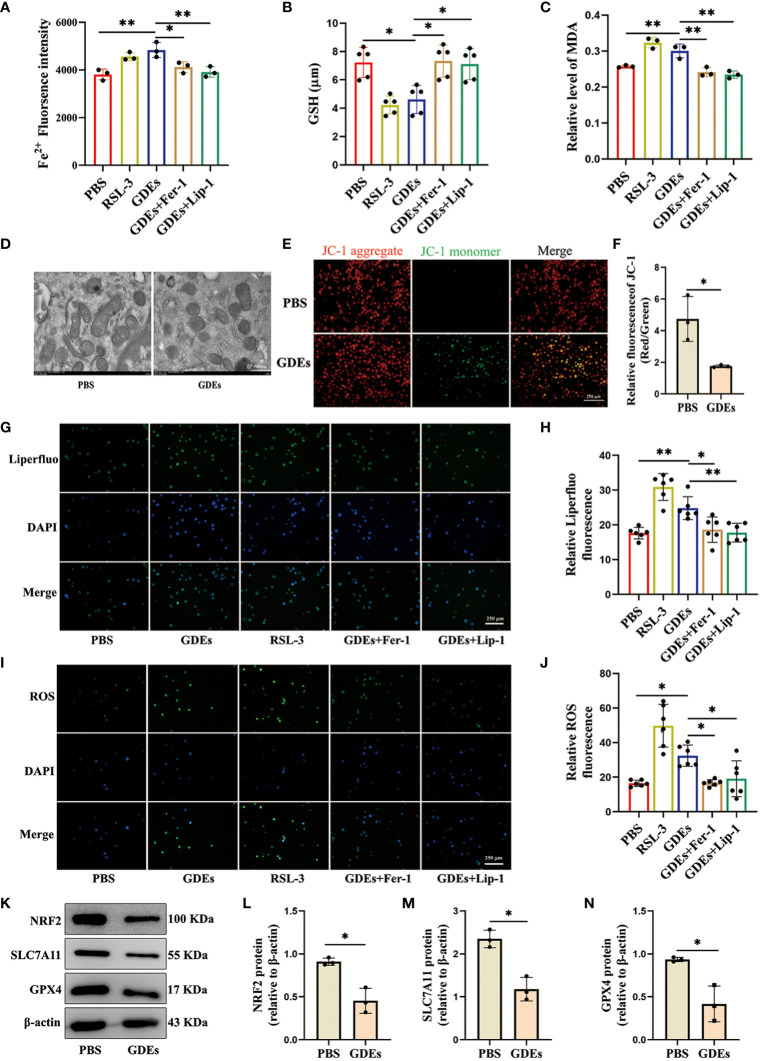
GDEs induced DCs ferroptosis vvia NRF2/GPX4 pathway. **(A)** mDCs treated with PBS, the GDEs (160 μg/mL), RSL3 (0.5 μg/mL), GDEs + Fer-1, and GDEs + Lip-1 for 24 h showed enhanced Fe^2+^ fluorescence intensity by zymography with n = 3. **(B)** GSH content changes in the PBS, GDEs, RSL3, GDEs + Fer-1, and GDEs + Lip-1 Groups 24 h after GDEs 160 μg/mL administration with n = 5. **(C)** The MDA content in the mDCs of different treatment groups was detected at 24 h with n = 3. **(D)** Transmission electron microscopy observed morphological changes in mitochondria with a decrease in mitochondrial bases. Scale bars, 500 nm. **(E, F)** JC-1 detects changes in mitochondrial membrane potential after 24h of GDEs treatment of mDCs with n = 3. Scale bars, 250 μm. ROS Reactive oxygen species, MDA Malondialdehyde, GSH Glutathione, RSL-3 ferroptosis inducer, Fer-1, Lip-1 ferroptosis inhibitor. **(G, H)** Fluorescence photographs were taken at 24 h and the fluorescence intensity of Liperfluo (lipid peroxidation) in mDCs was counted with n = 6. Scale bars, 250 μm. **(I, J)** Administration of GDEs at 160 μg/mL for 24 h showed an increase in intracellular ROS content with n = 6. Scale bars, 250 μm. **(K, N)** Western blot analysis of the PBS EXO group revealed NRF2 **(L)**, SLC7A11 **(M)**, GPX4 **(N)**, ferroptosis-related protein changes and bar charts are used for visualization with n = 3. Bars represent mean ± SD. *p<0.05; **p<0.01.

Given that previous report showed that excessive lipid accumulation and peroxidation in immune cells increases endoplastic reticulum stress in immune cells and suppresses their function (32). Lipid accumulation has been shown to lead to increased lipid peroxidation in cells (33). This is closely related to ferroptosis. Ferroptosis is a distinct form of cell death characterized by increased accumulation of lipid peroxidation (34, 35). To understand how GDEs induced lipid accumulation/peroxidadtion leads to increased cell death of DCs, We use ferroptosis inducer RSL3 (which inhibits GPX4 activity), the ferroptosis inhibitor Fer-1 (which eliminates excess free iron and reactive oxygen species within cells to reduce lipid peroxidation), and Lip-1 (activates the Nrf2 signaling pathway and restores GPX4 levels to inhibit ferroptosis) for intervention treatment (36, 37). We examined ferroptosis in mDCs after being treated with GDEs and found increased that Fe^2+^ content (**Figure 4A**), GSH (**Figure 4B**), and MDA levels (**Figure 4C**) in GDEs-treated mDCs compared to Control group. Furthermore, scanning electron microscopy analysis showed that GDEs treatment of DCs resulted in decreased mitochondrial volume, increased bilayer density, decreased mitochondrial cristae (**Figure 4D**) and a decrease of fluorescence intensities of mitochondrial membrane potential (**Figures 4E, F**). In addition, immunofluoscence analysis was used to measure lipid peroxide Liperfluo and ROS in each group. The data demonstrate that lipid peroxide Liperfluo and ROS levels were higher in the GDEs group compared with PBS group (**Figures 4G-J**). All these phenomena were inhibited by ferroptosis inhibitors. These results indicate that GDEs treatment contributes to ferroptosis in mDCs *in vitro*.

In the ferroptosis pathway, NRF2 is the major transcription factor that regulates SLC7A11 and GPX4 transcription (38). SLC7A11 is an ion channel protein that participates in the transport of cysteine. Cells use SLC7A11 to transport cysteine into the cell and participate in the synthesis of glutathione. Inhibition of SLC7A11 expression results in the suppression of GPX4 synthesis, which triggers ferroptosis (39). We conducted Western blot analysis to investigate the levels of ferroptosis-associated proteins SLC7A11, GPX4, and NRF2 in GDEs treated DCs and observed significant decrease of the levels of NRF2, SLC7A11 and GPX4 in GDEs treated DCs (**Figures 4K–N**).

### Reduction of GDEs secretion inhibited mDCs ferroptosis and tumor growth

**Figure 5:**
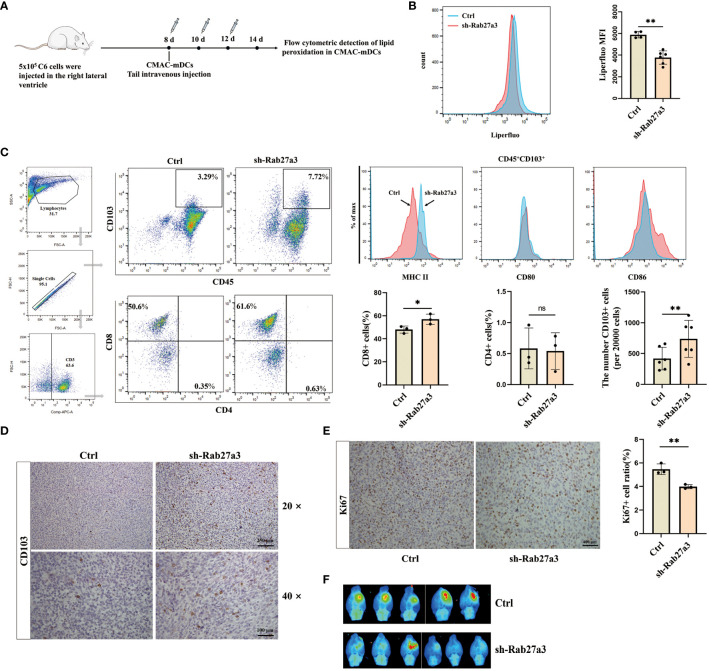
Reduction of GDEs secretion inhibited mDCs ferroptosis and tumor growth. **(A)** CMAC-mDCs injection and testing time flow chart. **(B)** Injection of CMAC-mDCs, detection of CMAC-mDCs lipid peroxidation in the Control and sh-Rab27a3 group by flow cytometry and bar charts are used for visualization. **(C)** Flow cytometric test the change of CD45^+^CD103^+^, CD4^+^, CD8^+^ cells and analysis MHCII/CD80/CD86 expression on the surface of CD45^+^CD103^+^ DCs in the Control and sh-Rab27a3 groups of glioblastoma models, as determined by flow cytometry assay at 14 d with n = 3 - 6. **(D)** CD103 DCs immunohistochemical staining in glioblastoma models from the Control and sh-Rab27a3 groups. Scale bars, 100 μm. **(E)** Conduct immunohistochemical staining for Ki67 on brain tissues of Control and sh-Rab27a3 glioblastoma models at 14 d, and determine the Ki67 positive rate. Scale bars, 250 μm. **(F)** On the 14 d, live imaging was conducted to observe the tumor size in the Control and sh-Rab27a3 group glioblastoma models. Bars represent mean ± SD. Cell experiment with n = 3, animals experiment per group with n = 5 – 6, *p<0.05; **p<0.01.

To investigate the effect of mDCs ferroptosis on glioblastoma growth. We cultured labeled bone marrow derived DCs with CellTracker Blue Dye (CMAC), and administrating CMAC-mDCs (5 × 10^5^ cells) from tail veins of rats at days 8, 10, and 12 after glioblastoma cells inoculation (**Figure 5A**). Subsequently, the levels of lipid peroxidation in CMAC-mDCs were measured using flow cytometric analysis at 14 d. The results demonstrated that the sh-Rab27a3 group had reduced ferroptosis in CMAC-mDCs compared to the Control group (**Figure 5B**). Meanwhile, we examined changes in the quantities of CD45^+^CD103^+^ DCs, CD3^+^CD8^+^ T, and CD3^+^CD4^+^ T cells in glioblastoma microenviroment. We observed that the sh-Rab27a3 group exhibited an increase in CD45^+^CD103^+^ DCs relative to Control group, which was further confirmed through CD103^+^ DCs immunohistochemistry (**Figures 5C, D**). The flow cytometric analysis showed that the proportion of CD3^+^CD8^+^ T cells was also increased in sh-Rab27a3 group compared to Control group, but no change was observed in CD3^+^CD4^+^ T cells (**Figure 5C**). Furthermore, we examined the expression of surface molecules MHCII, CD80, and CD86 in infiltrating CD45^+^CD103^+^ DCs in the Control and sh-Rab27a3 groups of glioblastoma. The results indicated elevated MHCII expression in the sh-Rab27a3 group, while CD80 and CD86 expression did not significantly change (**Figure 5C**). At the same time, immunohistochemistry results showed that the expression of Ki67, a proliferation marker in the glioblastoma area, was decreased in the sh-Rab27a3 group compared with the Control group (**Figure 5E**). *In vivo* imaging showed slow glioblastoma growth in sh-Rab27a3 group compared to Control group (**Figure 5F**). Overall, reduction of GDEs secretion inhibited mDCs ferroptosis and tumor growth.

## Conclusion

**Figure 6:**
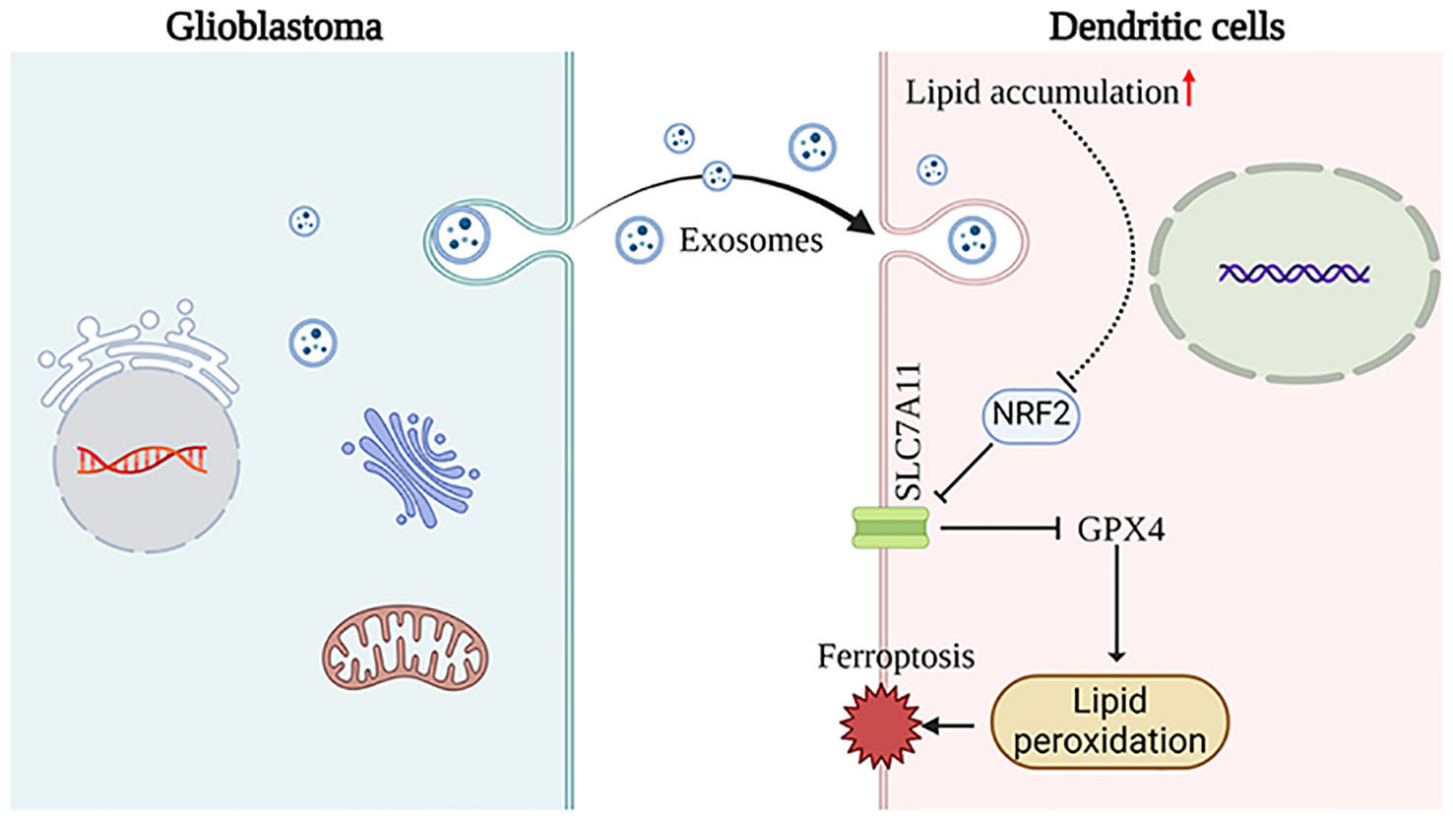
This schematic diagram illustrates the primary hypotheses and conclusions. GDEs are internalized by DCs, resulting in lipid accumulation. Subsequenty, the NRF2/GPX4 pathway promotes ferroptosis in mDCs. The images provided by https://www.biorender.com/.

These findings indicate that GDEs may induce ferroptosis in mDCs through the NRF2/GPX4 signaling pathway (**Figure 6**).

## Discussion

Immunotherapy for glioblastoma has advanced but faces challenges due to the tumor’s immunosuppressive microenvironment and its invasive growth pattern. Tumor-derived exosomes are known to modulate immune cell phenotypes by transferring proteins and genetic material. This study focuses on how GDEs influence mDCs, leading to lipid accumulation and ferroptosis. Our findings show that blocking exosome release reduces lipid content and peroxidation in DCs, and increases the number of MHCII^high^ DCs within the glioblastoma tissue in animal models. *In vitro*, GDEs treatment was found to enhance lipid uptake, accumulation, and peroxidation in mDCs, and to trigger ferroptosis via the NRF2/GPX4 pathway.

Glioma microenvironment contains various immune cells including T and B cells, macrophages, DCs, natural killer cells, neutrophils. GDEs may affect these adaptive and innate immune cells to exert their modulatory functions on these cells through various pathways. Indeed, our preliminary experiments showed that when peripheral blood mononuclear cells (PBMCs) were treated with PKH26-labeled GDEs, both macrophages and DCs showed strong uptake ability (**Supplementary Figure 2A**). Considering the characteristics of DCs in antigen presentation and T-cell activation, the expression of the antioxidant protein NRF2 in mDCs decreased compared to imDCs (**Supplementary Figure 2B**), we have chosen to study dendritic cells. Immune cells undergo ferroptosis during the process of fighting against tumors. Neutrophils, which possess important immune regulation and cytotoxic functions, experience ferroptosis resulting in immune suppression and tumor escape (40). CD8^+^ T cells are justified as they can promote lipid uptake through the CD36 molecule, which is necessary for ferroptosis to occur (41). Treg lymphocytes with GPX4 defects can induce ferroptosis during T cell receptor (TCR)/CD28 co-stimulation (41). Ferroptosis in mDCs results in their inability to induce CD8^+^ T cells to produce IFNγ and leading to decreased antigen presentation anti-tumor abilities in the tumor microenvironment. This occurrence of ferroptosis is likely caused by lipid accumulation (42, 43). In this study, we conducted a cellular study to investigate the effect of GDEs on ferroptosis in mDCs. Our research demonstrates that GDEs induce ferroptosis in mDCs cells by elevating Fe^2+^ levels, ROS, and lipid peroxides, simultaneously decreasing the protein expression of NRF2, SLC7A11, and GPX4.

Our research has revealed a correlation between GDEs and lipid accumulation in DCs infiltrating glioblastoma. However, our study merely treated GDEs as a whole and did not specifically investigate the types of lipids within GDEs. The specific reasons leading to the accumulation of lipid in DCs infiltrating glioblastoma have not been discussed in detail. We speculate that GDEs affect lipid accumulation and peroxidation levels in DCs may involve two pathways: (1) the direct transfer of fatty acids to DCs by GDEs; (2) activation of triglycerides endogenous synthesis pathway in DCs by GDEs. Excessive lipid accumulations within cells contribute to an increase in the level of lipid peroxidation. To investigate two pathways of the lipid accumulation in mDCs, we designed cell experiments that were intended to block key steps in the *de novo* synthesis pathway with two specific inhibitors, ACC (acetyl-coenzyme A [CoA] carboxylase) inhibitor 5-tetradecyloxy-2-furanone (TOFA) and fatty acid synthase inhibitor (C75). The results demonstrated that TOFA and C75 did not reduce the lipid content in DCs after GDEs treatment (**Figure 4O**). This indicates that direct transfer of fatty acids by GDEs might be main pathway of lipid accumulation in DCs. Further research is needed to elucidate the mechanisms by which GDEs transfer fatty acids and their impact on DCs’ own lipid metabolism.

In conclusion, our study demonstrated that GDEs induce lipid accumulation in DCs infiltrating glioblastoma, and leading to ferroptosis in mDCs through the NRF2/GPX4 signaling pathway. Our study will provide new ideas for DCs vaccine therapy with glioblastoma-loaded exosomes, and perhaps a DCs vaccine with reduced ferroptosis will have better efficacy.

## Data Availability

The original contributions presented in the study are included in the article/**Supplementary Material**. Further inquiries can be directed to the corresponding authors.
