# Supplementary material for: Glioblastoma-derived exosomes promote lipid accumulation and induce ferroptosis in dendritic cells via the NRF2/GPX4 pathway
Source: Front Immunol. 2024 Aug 13;15:1439191. doi: 10.3389/fimmu.2024.1439191 (PMC11347305; doi:10.3389/fimmu.2024.1439191)
Supplement: Supplementary file 1 [file DataSheet1.pdf]

Fig.1 sh-Rab27a3 did not affect the viability, invasion and migration of C6 cells in vitro.

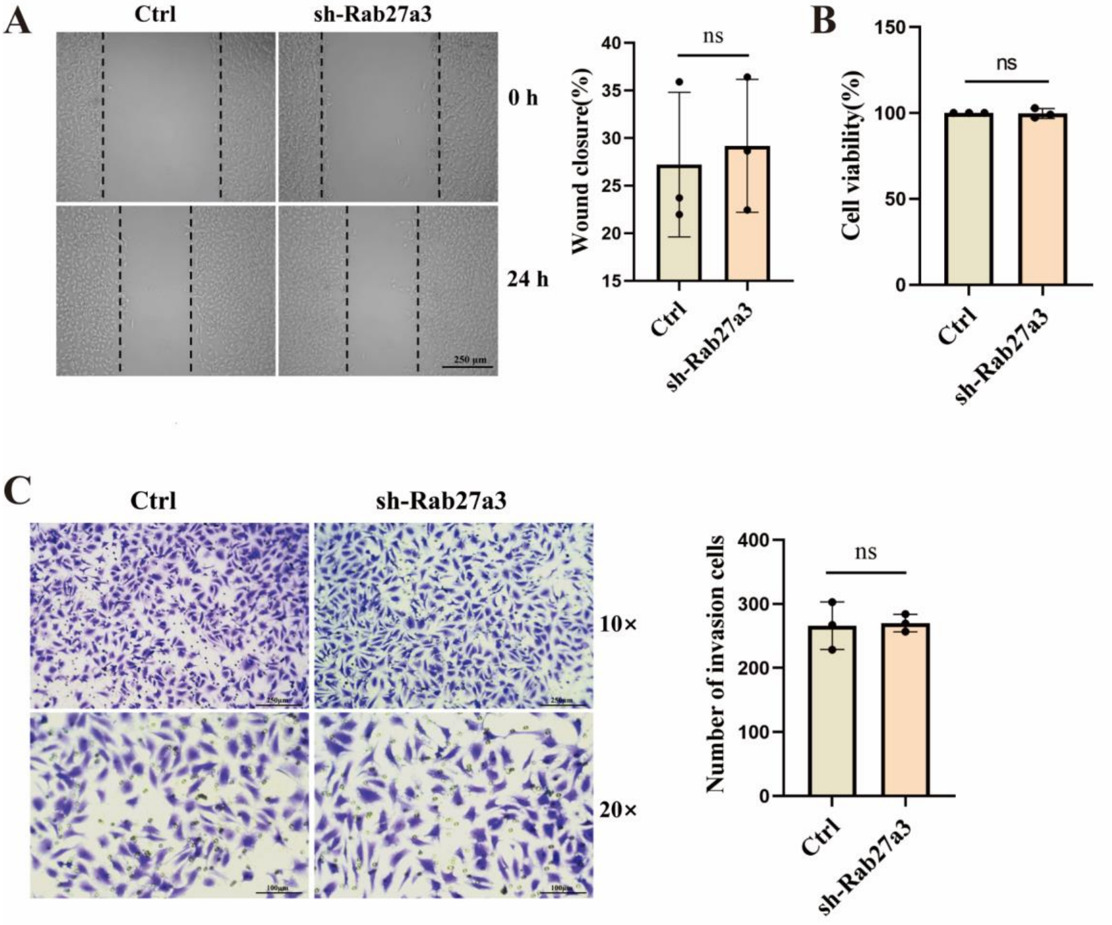

A Scratch assay was conducted to assess the migratory capability of C6 glioblastoma cells in both the 24 h control group and the sh-Rab27a3 group. Scale bars, 250 μm. **B** Cell viability of C6 glioblastoma cells was assessed using the CCK8 assay in both the control group and the sh-Rab27a3 group. **C** The invasive ability of both the 24h Ctrl group and sh-Rab27a3 group was assessed through the Transwell assay, the count of invasive cells was quantified. Scale bars, 100 μm.

Fig.2

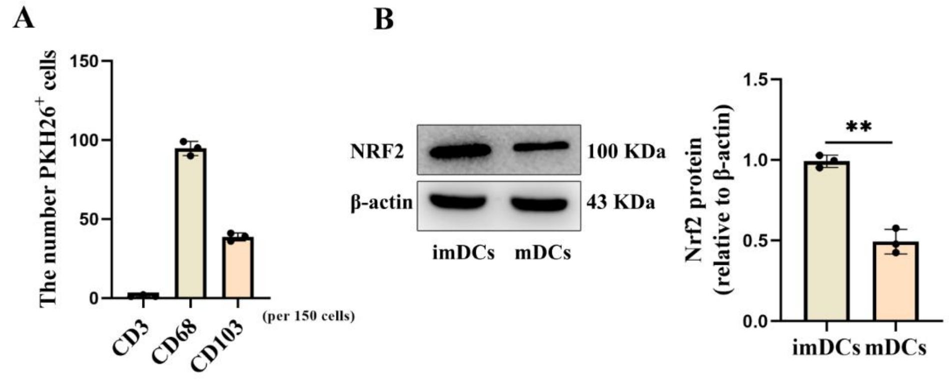

A PHK26-labeled GDEs to co-culture with PBMCs and assess the uptake capacity of CD3 T cells, CD68 macrophages, and CD103 dendritic cells. **B** Analysis the expression level of NRF2 protein in imDCs and mDCs.
